# Long-Term Clinical Outcomes of Gastric MALT Lymphoma: A Nationwide Multicenter Study in Korea

**DOI:** 10.3389/fonc.2021.681689

**Published:** 2021-10-14

**Authors:** Joon Sung Kim, Jun Chul Park, Jong Yeul Lee, Ji Yong Ahn, Sun Hyung Kang, Hyo-Joon Yang, Su Jin Kim, Moon Kyung Joo, Jae Myung Park

**Affiliations:** ^1^ Division of Gastroenterology, Department of Internal Medicine, Incheon St. Mary’s Hospital, College of Medicine, The Catholic University of Korea, Seoul, South Korea; ^2^ Division of Gastroenterology, Yonsei University College of Medicine, Severance Hospital, Seoul, South Korea; ^3^ Center for Gastric Cancer, National Cancer Center, Goyang, South Korea; ^4^ Division of Gastroenterology, Department of Internal Medicine, Asan Medical Center, University of Ulsan College of Medicine, Seoul, South Korea; ^5^ Division of Gastroenterology, Department of Internal Medicine, Chungnam National University School of Medicine, Dajeon, South Korea; ^6^ Division of Gastroenterology, Department of Internal Medicine and Gastrointestinal Cancer Center, Kangbuk Samsung Hospital, Sungkyunkwan University School of Medicine, Seoul, South Korea; ^7^ Division of Gastroenterology, Department of Internal Medicine, Pusan National University School of Medicine and Research Institute for Convergence of Biomedical Science and Technology, Pusan National University Yangsan Hospital, Yangsan, South Korea; ^8^ Division of Gastroenterology, Department of Internal Medicine, Korea University Guro Hospital, Korea University, College of Medicine, Seoul, South Korea; ^9^ Division of Gastroenterology, Department of Internal Medicine, Seoul St. Mary’s Hospital, College of Medicine, The Catholic University of Korea, Seoul, South Korea

**Keywords:** endoscopy, lymphoma, *Helicobacter pylori*, MALT, stomach

## Abstract

**Background:**

Treatment recommendations for gastric mucosa-associated lymphoid tissue (MALT) lymphoma are based on case series and expert opinions. Only a few previous studies have focused on the long-term outcomes of gastric MALT lymphoma, especially according to stage.

**Methods:**

Patients diagnosed with gastric MALT lymphoma from January 2000 to December 2018 at nine university hospitals in Korea were included. Clinical data of medical history, endoscopic features, histological diagnosis, results of *Helicobacter pylori* (*H. pylori*) testing, stage, treatment conditions, and outcomes were collected.

**Results:**

A total of 1,163 patients was enrolled, and 97.6% (n=1,038) of patients were diagnosed as stage IE. 10-year overall survival (OS) for the entire population was 99.1% and was better for patients in stage IE compared with patients in stage III/IV (*p*=0.002). The 10-year OS for *H. pylori*-positive patients was better than that of *H. pylori*-negative patients (*p*=0.022). Multivariate analyses revealed initial stage III/IV as a prognostic factor associated with over-all survival.

**Conclusion:**

The majority of gastric MALT lymphoma patients are diagnosed at an early localized stage in Korea. The overall survival rate of gastric MALT lymphoma is excellent and is associated with the initial stage of the disease.

## Introduction

Extranodal marginal zone B-cell lymphoma of mucosa-associated lymphoid tissue (MALT), also known as MALT lymphoma, arises from malignant transformation of B cells from the marginal zone of MALT (1). The gastrointestinal tract is the most common site of involvement in MALT lymphoma, with the stomach being the most common primary site (2, 3). Gastric MALT lymphoma is the most common type of gastric lymphoma, representing 38%-48% of primary gastric lymphoma cases (4–6). Gastric MALT lymphomas are generally low-grade lesions and are usually localized (7). The main risk of MALT lymphoma is its transformation to high-grade diffuse large B-cell lymphoma, although that is rare (8, 9).

*Helicobacter pylori* (*H. pylori*) plays a pivotal role in the pathogenesis of gastric MALT lymphoma. Epidemiologic studies show a close correlation between the prevalence of *H. pylori* infection and gastric lymphoma (10). *H. pylori* eradication is the treatment of choice in early stages, and successful eradication is sufficient for treatment in most cases (11, 12). Eradication therapy is recommended for early-stage disease, while radiation and surgery are reserved for advanced stages (13). Approximately 25% of patients do not respond to *H. pylori* eradication, and 20% of patients present as *H. pylori* negative (14, 15). The optimal management of these patients is not certain, and it is not clear if patients with more advanced disease should undergo eradication or additional treatment. However, the current treatment recommendations are based on case series and expert opinions (16, 17). Few studies have reported the treatment and survival of patients according to their different stages (18, 19). In addition, most previous studies that reported gastric MALT lymphoma survival rates included small population sizes. Understanding the survival rates of gastric MALT lymphoma patients according to stage should aid in improving therapeutic strategies. In this study, we evaluated treatments and long-term outcomes based on gastric MALT lymphoma stage in Korean patients. Specifically, we examined the survival rates of gastric MALT lymphoma patients according to stage and *H. pylori* infection status.

## Methods

### Patients

Patients diagnosed with gastric MALT lymphoma from January 2000 to December 2018, at nine university hospitals in Korea were enrolled. Medical records were reviewed retrospectively, and patients were excluded for any of the following conditions: synchronous cancer in another organ, no endoscopic follow-up, and/or incomplete medical records. The clinical data included medical history, endoscopic features, histological diagnosis, results of *H. pylori* testing, stage, treatment, and outcomes. The pathology reports were reviewed by an expert pathologist at each hospital, and the histological diagnosis was made according to the WHO Classification (20). The present study was conducted in accordance with the ethical guidelines of the Declaration of Helsinki and approved by the institutional review board of all participating hospitals.

### Diagnosis and Staging

Initial gastric MALT lymphoma diagnosis was based on endoscopic examination and histologic assessment of biopsy specimens. Gastric MALT lymphoma was diagnosed histologically by the presence of a diffuse infiltrate of centrocyte-like B cells in the lamina propria with prominent lymphoepithelial lesions formed by invasion of individual glands by aggregates of lymphoma cells under histologic examination (21). Location of the dominant lesion was grouped into the upper, middle, or lower third of the stomach, and clinical stage was determined using the modified Ann Arbor staging system (22). The staging work-up included physical examination, computed tomography (CT) scans of the chest and abdomen, and biopsies. Endoscopic ultrasound sonography (EUS), bone marrow aspiration, and chromosomal translocation t(11;18)(q21;q21) were assessed by the physician.

### *H. pylori* Infection and Eradication

*H. pylori* infection status was determined by histology, rapid urease test, or urea breath test, and *H. pylori* infection was regarded as positive when at least one of these tests yielded positive results. Eradication regimens consisted of standard triple therapy, sequential therapy, concomitant therapy, bismuth quadruple therapy, or levofloxacin-based triple therapy. The specific medication and dose for each regimen have been reported elsewhere (23). The outcomes of eradication therapy were determined using a urea breath test or a rapid urease test performed at least 4 weeks after completion of antibiotic therapy.

### Outcomes

The primary aim of this study was to examine the 10-year overall survival (OS) of gastric MALT lymphoma patients. We also examined the 10-year OS by stage, *H. pylori* infection status, and treatment. Clinical factors associated with OS also were assessed. OS was defined as the interval between the date of diagnosis and the final date of follow up and was censored at that date. We also calculated the median follow-up as the median time between diagnosis to the time the last subject has an event or is censored.

### Statistical Analysis

The baseline patient characteristics are summarized using descriptive statistics. The continuous data are presented as mean (standard deviation) or median (interquartile range) and categorical data as quantity and proportion. Comparison of categorical variables was performed using the χ^2^ test or Fisher’s exact test. The OS rates were calculated as the median time to censoring or death using Kaplan-Meier (KM) analysis and were compared using log-rank tests. The Cox proportional hazards model was applied to perform univariate and multivariate analyses to identify risk factors for mortality. Clinically relevant factors were included in the univariate model, whereas factors with *p* value less than 0.05 were considered for multivariate analyses. All statistical analyses were performed using SPSS software version 18.0 (SPSS Inc., Chicago, IL), and a *p* value less than 0.05 was considered statistically significant.

## Results

### Characteristics of Patients With Gastric MALT Lymphoma

**Figure 1:**
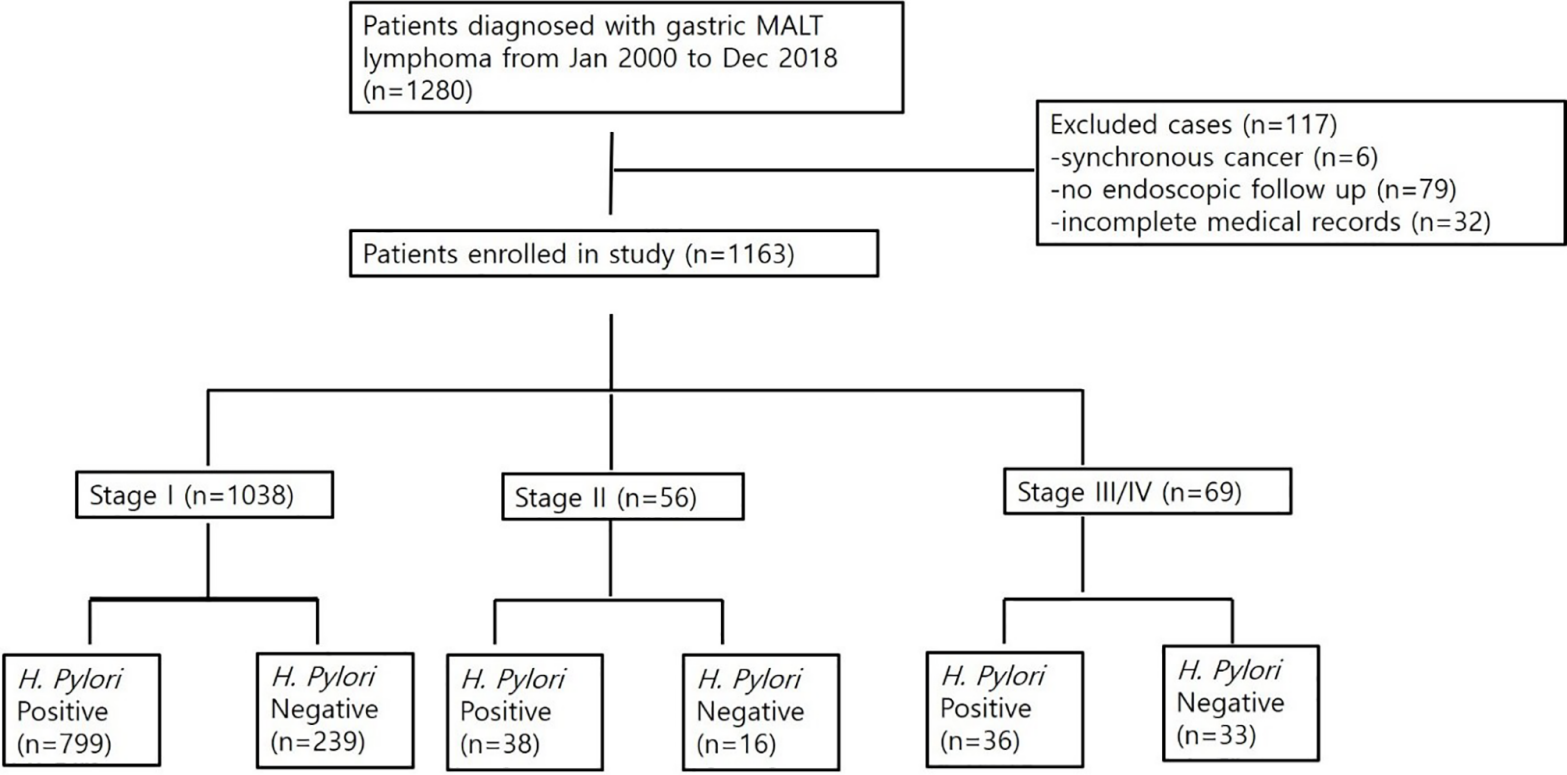
Flow chart of patients included in the study.

A total of 1,280 patients was diagnosed with gastric MALT lymphoma during the study period (**Figure 1**). Of these, 6 patients were excluded due to the presence of synchronous cancer, 79 patients were excluded due to lack of endoscopic follow up, and 32 patients were excluded due to incomplete medical records. Thus, 1,163 patients were included in the final analyses. The baseline characteristics of these patients are summarized in **Table 1**. The mean age at the time of diagnosis was 56 ± 12 years, and the proportion of males was 42.4%. The median follow-up for all patients was 102 months (range, 1.5 to 211 months). A total of 1,038 patients (97.6%) was diagnosed as stage IE, 56 patients (4.8%) were diagnosed as stage IIE, and 69 patients (5.9%) were diagnosed as stage III/IV. There was a significant difference in age, prevalence of *H. pylori*, and bone marrow involvement between the three groups. However, there was no difference in follow-up duration. Transformation into diffuse large B-cell lymphoma occurred in one patient diagnosed with stage IE and two patients diagnosed with stage II.

**Table 1:** Clinical characteristics of patients diagnosed with gastric MALT lymphoma.

	Stage Total	I (n = 1,038)	II (n = 56)	III/IV (n = 69)	*p-*value
Age, years	56 (12)	55.6 (11.9)	58.9 (14.3)	59.8 (10.5)	0.003
Male	493 (42.4)	428 (41.2)	34 (60.7)	31 (44.9)	0.015
*H. pylori* positivity*	873 (75.2)	799 (77.0)	38 (70.4)	36 (52.2)	<0.001
Location**					
Upper	374	321 (30.9)	20 (35.7)	33 (47.8)	
Middle	634	563 (54.2)	30 (53.63)	41 (59.4)	
Lower	386	352 (33.9)	17 (30.4)	17 (24.6)	
BM involvement***	30 (5.7)	0 (0)	0 (0)	30 (43.5)	<0.001
t(11;18)(q21;q21)****	10 (3.5)	7 (3)	0 (0)	3 (8.8)	0.228
Follow-up duration (months)	38 (23-62)	39 (23-61)	49 (24-67)	42 (22-58)	0.526

Data represent the number patients (%) or the mean (SD) or median (interquartile range).

*H. pylori infection status was not available for 2 patients.

**Patients had overlapping or multiple lesions.

***Bone marrow study was performed in 529 patients.

****Translocation t(11;18)(q21;q21) testing was performed in 288 patients.

### Treatment of Patients With Gastric MALT Lymphoma

**Table 2** shows the various treatments the patients received according to stage. *H. pylori* eradication was performed in 72.4% of stage IE patients and was the most frequent treatment method in this group. However, eradication was performed in 25.0% and 20.3% of stage IIE and stage III/IV patients, respectively. The most frequent treatment for stage IIE patients was *H. pylori* eradication, followed by radiotherapy, accounting for 35.7% of patients. The most frequent treatment for stage III/IV patients was chemotherapy, accounting for 29.0% of patients. However, *H. pylori* eradication followed by radiotherapy or chemotherapy also was frequent and accounted for 23.2% and 13.0% of patients, respectively.

**Table 2:** Treatment of patients with gastric MALT lymphoma according to stage.

	IE (n = 1,038)	IIE (n = 56)	IIIE & IV (n = 69)
Observation	26 (2.5)	3 (5.4)	1 (1.4)
Eradication	751 (72.4)	14 (25.0)	14 (20.3)
Eradication + CTx	12 (1.2)	8 (14.3)	16 (23.2)
Eradication + RTx	158 (15.2)	20 (35.7)	9 (13.0)
Initial CTx	13 (1.3)	3 (5.4)	20 (29.0)
Initial RTx	75 (7.2)	6 (10.7)	3 (4.3)
RTx+CTx	1 (0.1)	0 (0.0)	5 (7.2)
Eradication+RTx+CTx	2 (0.2)	2 (3.6)	1 (1.4)

CTx, chemotherapy; RTx, radiotherapy.

### Survival of Patients With Gastric MALT Lymphoma

**Figure 2:**
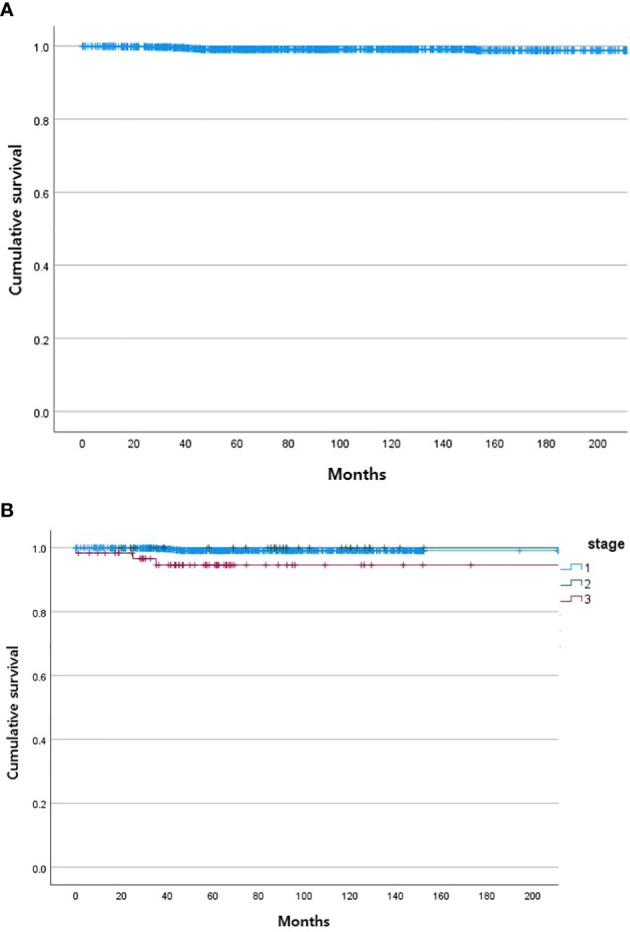
Kaplan Meier curves of gastric MALT lymphoma patient survival. **(A)** Total. **(B)** According to stage of gastric MALT lymphoma (stage I, stage II, stage III/IV).

**Figure 3:**
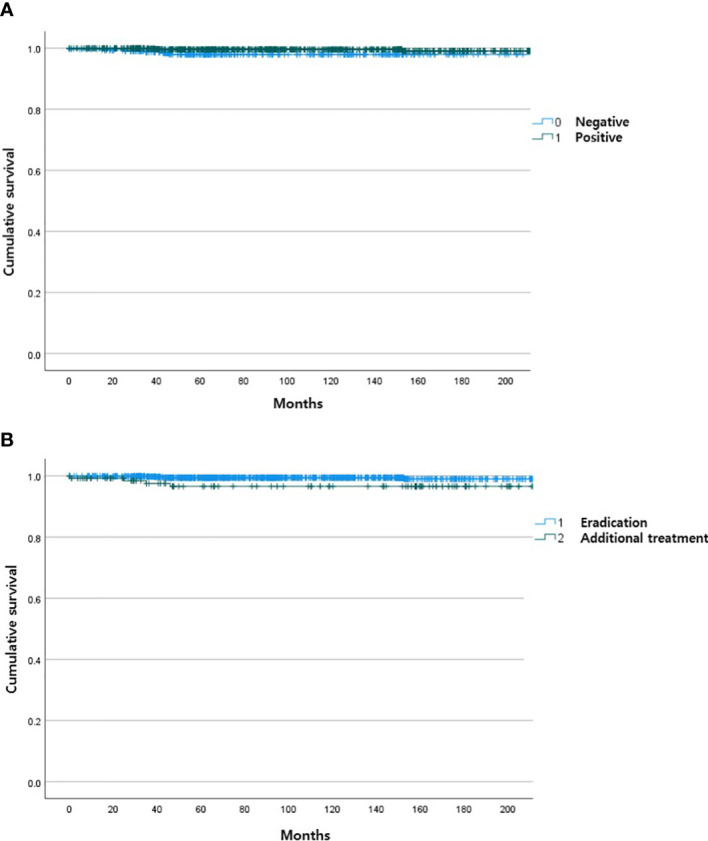
Kaplan Meier curves of gastric MALT lymphoma patient survival according to *H pylori* status **(A)** and type of treatment **(B)**.

During the follow-up period, 10 of the 1,163 patients died. The clinical features of the 10 patients who expired are shown in **Table 3**. The KM curves for 10-year OS are presented in **Figure 2**. The 10-year OS for the entire population was 99.1% (**Figure 2A**), and 10-year OS for stage IE, IIE, and III/IV patients were 99.3%, 100%, and 94.6%, respectively (**Figure 2B**). 10-year OS was better for stage IE patients compared to stage III/IV patients (*p*=0.002). The 10-year OS for patients who were *H. pylori* positive was 99.5%, which was better than the 97.9% for *H. pylori*-negative patients (*p*=0.022, **Figure 3**). The 10-year OS rates of patients who received only *H. pylori* therapy was 99.5% and was better than the 96.6% of that of patients who received additional treatment after eradication or other treatment (*p*=0.007). The multivariate model adjusted for treatment showed initial stage III/IV as a poor prognostic factor (**Table 4**).

**Table 3:** Summary of patients who died after gastric MALT lymphoma diagnosis.

Sex	Age	*H. pylori* status	Stage	Initial treatment	Time to death from diagnosis (months)	Cause of death
M	62	Negative	IV	CTx	51	Septic shock after chemotherapy
M	57	Negative	IE	Eradication	14	Hepatic failure
F	63	Negative	III	CTx	21	Septic shock after chemotherapy
F	63	Positive	IE	Eradication	137	Pneumonia
M	57	Negative	IV	CTx	12	Pneumonia
M	60	Positive	IE	Eradication	41	Pneumonia
F	41	Negative	IE	Eradication	91	Pancreatic cancer
F	65	Positive	IE	Eradication	44	Pneumonia
F	50	Negative	IE	CTx	95	Breast cancer
F	45	Positive	IE	Eradication	51	Gastric cancer

H. pylori, Helicobacter pylori.

**Table 4:** Univariate and multivariate analyses of clinical factors associated with overall survival.

Variable	Univariate analysis		Multivariate analysis	
	HR (95% CI)	*p-*value	HR (95% CI)	*p*-value
Age	1.006 (0.955-1.060)	0.816		
Male sex	1.028 (0.290-3.649)	0.965		
*H. pylori* positivity	0.228 (0.064-0.811)	0.022	0.297 (0.080-1.102)	0.070
BM involvement	4.585 (0.511-41.137)	0.174		
t(11:18)(q21;q21)	0.46 (0.000-9346.707)	0.840		
Stage III/IV	8.872 (2.195-35.858)	0.002	6.197 (1.466-26.184)	0.013

HR, hazard ratio; CI, confidence interval; BM, bone marrow.

## Discussion

The main stay of treatment for low-grade gastric MALT lymphoma is *H. pylori* eradication. A systematic review of 1,408 patients with low-grade lymphoma at an early stage found that *H. pylori* eradication achieved remission in 77.5% of patients (12). Earlier studies reported that 5-year OS and disease-free survival (DFS) rates were as high as 90% and 75%, respectively, when lymphoma was treated at an early stage (24). However, a recent population-based study in France reported 5-year OS rates of 79%, which is considerably lower than that of previous studies (18). This study reported 25% of patients diagnosed at an advanced stage (III/IV), which is in contrast to most previous clinical studies that reported greater than 90% of patients with gastric MALT lymphoma diagnosed at a localized stage (25, 26). The larger percentage of patients in stage III/IV might have resulted in a lower survival rate in this study compared to previous studies.

The 10-year OS of gastric MALT lymphoma patients in our study was 99.1% and considerably higher than that reported previously. Korea has a high prevalence of *H. pylori* and shows the highest age-standardized incidence rate of gastric cancer worldwide (27, 28). A National Cancer Screening Program (NCSP) for gastric cancer invites every Korean individual 40 years of age or older to undergo endoscopy or an upper gastrointestinal series every 2 years. Participation rates for the NCSP increased from 7.5% in 2002 to 47.3% in 2012 (29). Gastric MALT lymphoma frequently is found incidentally at an early stage during screening endoscopy for gastric cancer in Korea. This is evidenced by the high rates of stage IE disease in our study.

Approximately 25% of patients do not respond to *H. pylori* eradication, and 20% of patients present with *H. pylori*-negative gastric MALT lymphoma (14, 15). The optimal management of these patients is not certain, and there is no consensus regarding optimal management of advanced gastric MALT lymphoma that has spread to lymph nodes or other extranodal sites. Current guidelines recommend *H. pylori* eradication as the first-line treatment of choice for all gastric MALT lymphomas (30). Our study showed great heterogeneity in treatment modalities and nonadherence to current guidelines. Surprisingly, some patients with localized stages received overtreatment with systematic therapy. Heterogeneity was the greatest in patients with advanced stage gastric MALT lymphoma. *H. pylori* eradication was performed in only 58% of stage III/IV patients. Chemotherapy and radiation was performed in 29.0% and 4.3% of patients. Despite the various treatments, the OS of advanced stage patients was still high in our study. Future studies are warranted to determine the best treatment modality for this group of patients. Efforts should be made to increase general awareness and adherence to current practice guidelines (16, 17, 31).

Recently, a MALT lymphoma prognostic index was developed from a Cox regression analysis of 401 patients (32). The authors reported age, Ann Arbor stage III or IV, and an elevated lactate dehydrogenase level as the main factors associated with survival. This prognostic index was derived from a heterogeneous group of patients including both gastric and non-gastric lymphomas. In our study, we examined the factors associated with survival in only gastric MALT lymphoma patients and found stage III/IV of the disease as the only factor associated with OS.

This study has limitations, most of which are inherent to its retrospective nature. First, there was a wide variation in treatments among patients, which only allowed us to estimate the OS of patients according to stage. The different types of treatments and their possible implications in the survival of patients need to be assessed in the future. *H. pylori* infection may have been underestimated in our study as the diagnostic methods were not predetermined. We also cannot exclude that the results from our study are influenced by confounding factors due to the observational nature of our study design; furthermore, risk of selection bias cannot be overlooked. Our study reported a high rate of 10-year OS compared to previous studies, even for advanced-stage gastric MALT lymphomas. We also did not find any disease related deaths associated with gastric MALT lymphomas which is contrary to previous reports (18). However, our results are consistent with those of a recent study that examined the survival rates of MALT lymphoma from the Korean Center Cancer Registry (33). This study reported survival rates of 97.9% for unknown stages of MALT-lymphoma during the 2013-2017 period. To the best of our knowledge, our study examined the largest number of gastric MALT lymphoma patients over a long follow-up period. We argue that our multi-center study is a fairly accurate illustration of routine clinical practice and outcomes of gastric MALT lymphoma despite its retrospective design.

In conclusion, the majority of gastric MALT lymphoma patients is diagnosed at an early stage in Korea. The OS of gastric MALT lymphoma is excellent and is associated with the initial stage of the disease. Future studies are warranted to determine the best treatment modality in patients with advanced-stage disease.

## Data Availability Statement

The raw data supporting the conclusions of this article will be made available by the authors, without undue reservation.

## Ethics Statement

The studies involving human participants were reviewed and approved by IRB of Catholic University of Korea. The ethics committee waived the requirement of written informed consent for participation.

## Author Contributions

JK and JCP: planned the study. JK, JCP, JL, and JA wrote the manuscript. All authors interpreted the date, revised the manuscript for intellectual content, and approved the final manuscript.

## Funding

This research was supported by the 2020 Research Fund from the Korean College of Helicobacter and Upper Gastrointestinal Research. (KCHUGR-20200250).

## Conflict of Interest

The authors declare that the research was conducted in the absence of any commercial or financial relationships that could be construed as a potential conflict of interest.

The reviewer EJG declared a shared affiliation, with one of the authors JA to the handling editor at the time of the review.

## Publisher’s Note

All claims expressed in this article are solely those of the authors and do not necessarily represent those of their affiliated organizations, or those of the publisher, the editors and the reviewers. Any product that may be evaluated in this article, or claim that may be made by its manufacturer, is not guaranteed or endorsed by the publisher.
